# Poster Session I - A118 TREATMENT OF IDIOPATHIC DIABETIC DIARRHEA: A SYSTEMATIC REVIEW

**DOI:** 10.1093/jcag/gwaf042.118

**Published:** 2026-02-13

**Authors:** A Chang, R S Cho, T Wai, B Nguyen

**Affiliations:** Medicine, The University of British Columbia, Vancouver, BC, Canada; University of Waterloo, Waterloo, ON, Canada; Medicine, The University of British Columbia, Vancouver, BC, Canada; Medicine, The University of British Columbia, Vancouver, BC, Canada

## Abstract

**Background:**

Idiopathic diabetic diarrhea (IDD) is a complication of diabetes mellitus, marked by chronic loose or watery bowel movements without an identifiable cause. IDD remains poorly understood in the literature, with limited evidence on effective management.

**Aims:**

To assess the scope of current literature on available treatments for IDD.

**Methods:**

We conducted a systematic review across 4 databases, following PRISMA guidelines. Cases with known pathology, non-primary, non-human, or inadequate treatment data were excluded. Descriptive statistics were performed after data extractions outlining study designs, patient traits, methods of diabetes management, treatments, and symptomatic outcomes. Absence of randomized controlled trials and heterogeneous outcome measures made meta-analysis unfeasible.

**Results:**

21 studies (16 case reports, 5 case series) from 1960-2013 met the inclusion criteria (Table 1). Somatostatin analogues demonstrated the highest improvement in symptoms (92.3%; 12/13). Despite smaller sample sizes, 5-HT3 antagonists (75.0%, 3/4), corticosteroids/immunomodulators (66.7%, 2/3), and alpha-2 adrenergic agonists also showed response. Opiate antidiarrheal agents were most frequently prescribed, but only 22.9% (8/35) experienced improvement. Anticholinergics/antispasmodics (40.0%, 2/5), bile acid sequestrants (33.3%, 3/9), dietary interventions (23.8%, 5/21), and antibiotics (21.7%, 5/23) yielded variable or modest responses. Other therapies without symptomatic response included prokinetic agents, pancreatic enzyme therapy, and NSAIDs.

**Conclusions:**

Among available cases, symptoms most often improved with somatostatin analogues. 5-HT3 antagonists, corticosteroids, and alpha-2 adrenergic agonists were less common but showed similar rates. Opiate antidiarrheal agents, antibiotics, and nutritional interventions were frequently prescribed despite low rates of success. The availability of certain treatments may have been influenced by year and country of origin. As literature was limited to case reports and case series, larger studies and more recent data are necessary for better identification and formal comparison of treatment.

**Funding Agencies:**

None

**A118 Table 1:** Summary of Findings on Treatments of Idiopathic Diabetic Diarrhea

Treatments	Total Cases	Positive Cases (Improved Symptoms)	Success Rate (Positive Cases/Total Cases)
Opiate Antidiarrheal Agents	35	8	22.86%
Antibiotics	23	5	21.74%
Nutritional Interventions	21	5	23.81%
Somatostatin Analogues	13	12	92.31%
Alpha-2 Adrenergic Agonists	9	5	55.56%
Bile Acid Sequestrants	9	3	33.33%
Pancreatic/Digestive Enzymes	9	0	0.00%
Prokinetic/Motility Agents	7	0	0.00%
Anticholinergic/Antispasmodic Agents	5	2	40.00%
5-HT3 Antagonists	4	3	75.00%
Corticosteroids/Immunomodulators	3	2	66.67%
Anti-inflammatory/NSAIDs	1	0	0.00%

